# Anti-Hyperglycemic Effects of Green Crab Hydrolysates Derived by Commercially Available Enzymes

**DOI:** 10.3390/foods9030258

**Published:** 2020-02-28

**Authors:** Bouhee Kang, Denise I. Skonberg, Angela D. Myracle

**Affiliations:** 1 School of Food and Agriculture, University of Maine, 5735 Hitchner Hall, Orono, ME 04469, USA; bouhee.kang@maine.edu; 2 School of Food and Agriculture, University of Maine, 5763 Rogers Hall, Orono, ME 04469, USA; angela.myracle@maine.edu

**Keywords:** green crab, protein hydrolysates, enzymatic hydrolysis, type 2 diabetes, anti-hyperglycemia

## Abstract

The predation and burrowing activity of invasive green crabs have had detrimental effects on important marine resources and habitats. Our objective is to develop bioactive hydrolysates by enzymatic proteolysis of underutilized green crab. Mechanically separated mince was hydrolyzed with Alcalase, Protamex, Flavourzyme, and Papain (1%) for 60 min. Subsequently, the hydrolysates were introduced to a simulated gastrointestinal digestion model. Selected samples were fractionated by ultrafiltration, and their anti-hyperglycemic effects including α-glucosidase, α-amylase, and dipeptidyl peptidase-IV (DPP-IV) inhibitory activities and glucagon-like 1 (GLP-1) secretory activity were evaluated. The Protamex treatment showed the highest α-glucosidase inhibitory activity (IC_50_ 1.38 ± 0.19 mg/mL) compared to other enzyme treatments and the crab mince control, and its α-amylase inhibitory activity (IC_50_ 11.02 ± 0.69 mg/mL) was lower than its α-glucosidase inhibitory activity. Its GLP-1 secretory activity was approximately four times higher than the positive control (10 mM glutamine). The <3 kD fraction contributed significantly to the anti-hyperglycemic activity of Protamex-derived hydrolysates, and this activity was stable after simulated digestion. Our results suggest that green crab hydrolysates obtained by Protamex treatment have the potential for type 2 diabetes management and could be incorporated in food products as a health-promoting ingredient.

## 1. Introduction

In recent years, consumer interest in sustainability and healthy lifestyles has significantly increased, leading to higher demand for nutritious and environmentally-friendly food products among consumers and the food industry. The development of natural, bioactive food ingredients from currently underutilized food resources may positively contribute to satisfying this demand. Nutritional and bioactive food ingredients include proteins and peptides, polyunsaturated fatty acids (PUFAs), phytochemicals, fibers, probiotics, and prebiotics [1]. Marine organisms represent good sources for obtaining various bioactive compounds, and many of these bioactive compounds are derived from proteins [2]. Therefore, protein-rich marine organisms are an ideal raw starting material for the development of protein-derived bioactive compounds.

European green crabs (*Carcinus maenas*) are an invasive species well established in the U.S. that negatively influence fisheries, aquaculture, and marine habitats due to their high resilience, voracious predation, and strong burrowing activity [3,4,5,6]. Green crabs are very competitive predators that prefer juvenile clams, blue mussels, and lobster larvae, which are highly profitable for fisheries and aquaculture, as their prey [3,4]. Their intensive burrowing and high viability have destroyed not only valuable eelgrass beds and salt marshes but also the overall balance of the natural marine ecosystem [5]. Although there is a large population of green crabs in the U.S., they are not currently utilized by food industries due to their small size. To obtain the nutritious meat, significant labor and high costs are required to separate the muscle from the carapace. However, our previous studies demonstrated that green crab mince obtained via mechanical separation can be used in the development of value-added food products [7,8].

Green crab contains various nutritional and nutraceutical compounds such as chitin, carotenoids, essential amino acids, and PUFAs including eicosapentaenoic acid (EPA) and docosahexaenoic acid (DHA) [9]. Green crab meat has approximately 80% protein [10]; these proteins have the potential to be utilized as the initial material for the generation of multifunctional food ingredients. Some proteins and peptides extracted from protein-rich marine resources have been reported to act as functional and bioactive ingredients in food products [11,12]. Enzymatic hydrolysis of a variety of protein sources has been extensively utilized as a method to generate bioactive peptides having antioxidant, anti-inflammatory, and anti-hypertensive effects [13,14,15]. Different types of proteases used for enzymatic hydrolysis have distinct specificity and produce peptides having different molecular weights and amino acid sequences, which may contribute to their diverse biological and physiochemical functional properties [16,17,18]. Various studies have confirmed that commercially available proteases such as Alcalase, Protamex, Flavourzyme, and Papain have the potential to generate bioactive peptides from food proteins [19,20]. However, the majority of these studies were conducted with dairy proteins and finfish byproducts. Furthermore, the bioactivity of hydrolysates obtained from marine resources was studied extensively with regard to their anti-hypertensive and antioxidant activities rather than their anti-diabetic properties. Fewer studies have been conducted to assess the biofunctional activity of the valuable peptides derived from shellfish proteins. More specifically, there is a lack of research about the potential anti-diabetic activity of crustacean and mollusk proteins and their hydrolysates.

Type 2 diabetes mellitus (T2DM) is one of the most prevalent chronic diseases in the world and ranks in the top 10 diseases causing the most deaths worldwide (WHO) [21]. T2DM and associated complications result not only in health issues but also substantial direct and indirect costs [22]. However, an optimal diet can help in the prevention of T2DM and is critical to reducing the attendant morbidity and cost associated with the treatment of T2DM [23,24,25]. The development of anti-hyperglycemic ingredients from green crabs may contribute to the prevention of T2DM and reduce the use of pharmaceutical products and the risks associated with their side-effects [26]. Furthermore, the development of natural and nutraceutical peptides generated from green crabs may encourage the development of a fishery for this invasive predator. Therefore, the aim of our study was to investigate the anti-hyperglycemic effect of green crab hydrolysates for potential use as a health-promoting food ingredient. To achieve this, (1) enzymatic hydrolysis using commercially available proteases was applied to mechanically separated green crab mince, (2) the potential anti-hyperglycemic effects of green crab hydrolysates were investigated, (3) fractionation of hydrolysates using ultrafiltration was conducted, and (4) changes in anti-hyperglycemic activity of green crab hydrolysates after simulated gastrointestinal digestion were investigated.

## 2. Materials and Methods

### 2.1. Materials and Reagents

Green crabs (carapace width: 40–85 mm and weight: 20–155 g) were harvested in 2018 on the Back River in Georgetown, Maine, USA. All of the chemicals and reagents that were used in this study were supplied by Fisher Scientific (Hampton, NH, USA), Thermo Fisher Scientific (Waltham, MA, USA), Bio-Rad Laboratories (Hercules, CA, USA), and Sigma-Aldrich (St. Louis, MO, USA) unless otherwise described. Alcalase 2.4 L (AL), Protamex (PR), Flavourzyme (FL), and Papain (PA) were purchased from Sigma-Aldrich (St. Louis, MO, USA). Rat intestinal acetone powders of α-glucosidase (EC 3.2.1.20), porcine pancreatic α-amylase (EC 3.2.1.1), pepsin from porcine gastric mucosa (EC 3.4.23.1, >250 units/mg protein), pancreatin from porcine pancreas (EC 232.468.9, 8 × USP), p-nitrophenyl-β-D-glucopyranoside (p-NPG), and Gly-Pro-p-nitroanilide were purchased from Sigma-Aldrich (St. Louis, MO, USA). Porcine kidney dipeptidyl peptidase-IV (DPP-IV) (E.C. 3.4.14.5) and glucagon-like 1 (GLP-1) ELISA kit were purchased from EMD Millipore (Billerica, MA, USA). Cell culture materials including penicillin-streptomycin (0.1g/L), Dulbecco’s modified Eagle’s medium (DMEM) containing low glucose, Glutamax, trypsin/EDTA (10×), and Poly D-lysine were obtained from Gibco Life Technologies (Grand Island, NY, USA). Fetal bovine serum (FBS) was purchased from Atlanta Biologicals (Minneapolis, MN, USA). Bovine serum albumin, acarbose, sitagliptin, and glutamine were obtained from Sigma-Aldrich (St. Louis, MO, USA).

### 2.2. Preparation of Green Crab

**Figure 1:**
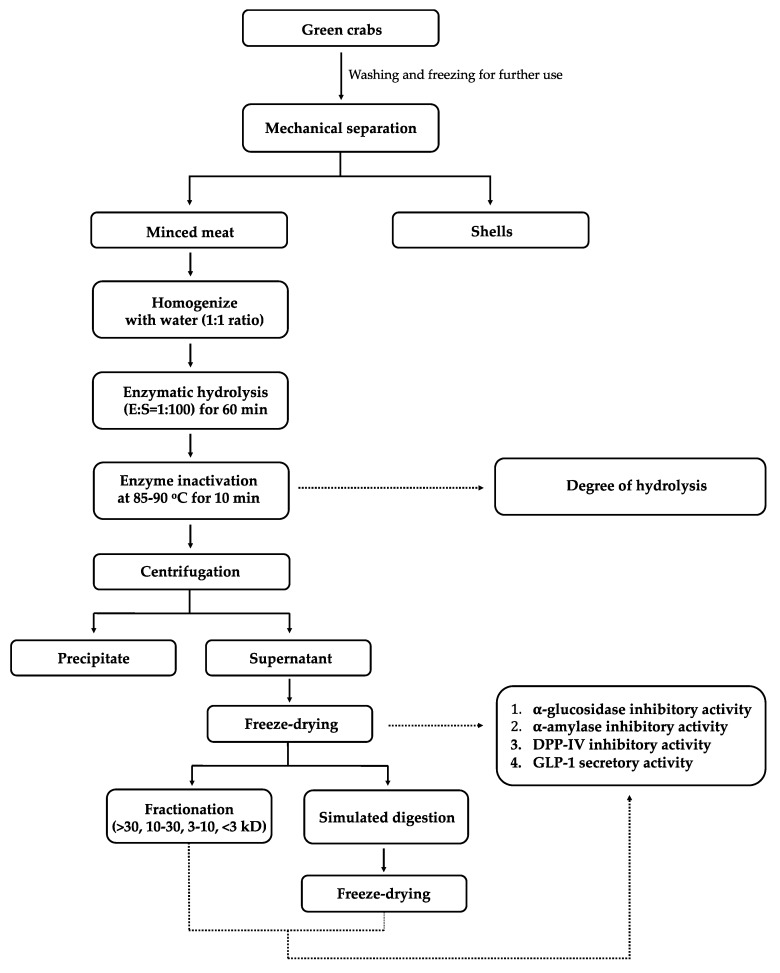
Process flow of sample preparation.

Forty kilograms of harvested crabs were washed with tap water and then blast-frozen at −30 °C for 1 h. Frozen crabs were stored at −20 °C until further use. Partially thawed green crabs were processed through a mechanical separator (Paoli One-Step mechanical separator, Rockford, IL) to separate meat and shell streams, and then the minced meat was vacuum sealed in plastic bags (3 mil, 3.3 cm^3^/100 in^2^ oxygen transmission rate, 80 microns, UltraSource, Kansas, MO, USA). The crab mince was blast-frozen at −30 °C for 1 h and then stored at 20 °C until further use (Figure 1).

### 2.3. Enzymatic Hydrolysis of Crab Mince

Enzymatic hydrolysis using commercially available proteases was conducted based on a modified protocol from Beaulieu et al. [27]. The raw crab mince was mixed with deionized water in a 1:1 (*w*/*w*) ratio, then homogenized for 1 min in a Waring blender at maximum speed. The enzymatic hydrolysis was conducted at the optimum pH and temperature of each enzyme including Alcalase (AL, 50 °C and pH 8), Protamex (PR, 50 °C and pH 7), Flavourzyme (FL, 50 °C and pH 7), and Papain (PA, 65 °C and pH 6). Protein content was determined based on total nitrogen content as analyzed by a combustion analyzer (TRU MAC CNS, LECO Corp., St. Joseph, MI, USA). Protein content was determined using a protein conversion factor of 6.25. Enzyme was added to the homogenate based on substrate (protein) content (1:100 = E:S), and the mixture was hydrolyzed for 60 min. During hydrolysis, the pH of the mixture was maintained using 6 N HCl or 6 N NaOH and temperature was monitored. After hydrolysis, the mixture was heated at 85–90 °C for 10 min to inactivate the enzyme and then immediately cooled on ice. For the crab mince control (CMC), mince was homogenized with water but not subsequently treated with the enzyme. Subsampling was performed to evaluate the degree of hydrolysis, and then the mixtures were centrifuged at 19,722× *g* for 15 min at 4 °C, and the supernatants were collected. All of the treatments were processed in triplicate. The collected supernatants were blast-frozen at −30 °C for 1 h, then freeze-dried (35 EL, VirTis Co. Inc., Gardiner, NY, USA) at −30 to 25 °C under 250 mT for 10 days All lyophilized supernatants were stored at −80 °C until further use.

### 2.4. Degree of Hydrolysis

Degree of hydrolysis was determined following the O-phthalaldehyde (OPA) method [28,29]. OPA reagent was prepared with 375 mL of deionized water, 19.05 g of sodium tetraborate decahydrate, 500 mg of sodium dodecyl sulfate (SDS), and 400 mg of 97% OPA in 10 mL of ethanol. After mixing, 440 mg of 99% dithiothreitol (DTT) was added to the solution and deionized water was added to achieve a final volume of 500 mL. For the sample preparation, the CMC and enzyme hydrolysates were diluted with 4% SDS (1:19 *w*/*v*). After centrifugation at 1100× *g* for 10 min, 4 mL of supernatant was collected. Subsequently, the supernatant was diluted to 50 mL with deionized water. Four mL of OPA reagent were mixed with 400 µL of solubilized sample/standard (0.5 mg/mL serine) and the mixture was incubated at room temperature for 2 min. Absorbance was measured at 340 nm and the degree of hydrolysis was calculated based on the following three equations:

(1) $${\mathrm{Serine-NH}}_{2}\;\left(\mathrm{mequiv}/g\;\mathrm{protein}\right)=\frac{\frac{\left({\mathrm{OD}}_{\mathrm{sample}}-{\mathrm{OD}}_{\mathrm{blank}}\right)}{\left({\mathrm{OD}}_{\mathrm{standard}}-{\mathrm{OD}}_{\mathrm{blank}}\right)}\times 0.9516\frac{\mathrm{mequiv}}{L}\times 0.05\times 100}{W\times \frac{V1}{V2}\times P}$$

W = weight of sample (g);

P = protein (g/100 g sample);

V1 = volume of supernatant (4 mL);

V2 = volume of SDS solution (20 mL);

0.05 = sample volume in L.

(2) $$h=\frac{\left(\mathrm{Serine}-\mathrm{NH}2\right)-\beta }{\alpha }$$

h = number of hydrolyzed peptide bonds;

α = 1.00, β = 0.40 mequiv/g protein;

(3) $$\mathrm{DH}=\frac{h}{h_{\mathrm{total}}}\times 100$$

Total number of peptide bonds (h_total_) of fish: 8.6 mequiv/g protein.

### 2.5. Simulated Gastrointestinal Digestion

Simulated gastrointestinal digestion of the control and crab mince hydrolysates was performed according to González-Montoya et al. [30]. The sample was dissolved in deionized water (5% *w*/*v*), then pH was adjusted to pH 2 using 6 N HCl prior to heating to 37 °C. Pepsin was added to the sample mixture (4:100 = E:S protein/peptide content) and stirred at 37 °C for 60 min. Then, pH was adjusted to 7.5 using 6 N NaOH. Subsequently, pancreatin (4:100 = E:S protein/peptide content) was added for the intestinal digestion phase and the mixture was stirred at 37 °C for 2 h. To inactivate the digestive enzymes, the mixture was heated at 85–90 °C for 10 min. The mixture was freeze-dried (35 EL, VirTis Co. Inc., Gardiner, NY, USA) at −30 to 25 °C under 250 mT for 10 days and then stored at −80 °C until further use.

### 2.6. Sodium Dodecyl Sulfate Polyacrylamide Gel Electrophoresis (SDS-PAGE)

Molecular weight distribution was determined by SDS-PAGE. All of the samples were dissolved in a Laemmli sample buffer containing 62.5 mM Tris-HCl (pH 6.8), 2% SDS, 25% (*v*/*v*) glycerol, 0.01% bromophenol blue, and 5% β-mercaptoethanol, then heated in a 90–95 °C water bath for 5 min [31]. Samples were separated on a 4% stacking and 16.5% separating gel (Bio-Rad Laboratories, Hercules, CA) using Tris/Glycine/SDS running buffer (25 mM Tris, 192 mM glycine, 0.1% (*w*/*v*) SDS) in a Bio-Rad Mini-PROTEAN III Cell (Bio-Rad Laboratories, Inc., Hercules, CA, USA). The amount that was loaded to the wells was adjusted to 1 mg for the hydrolysates, 0.5 mg for the control, and 10 µL of a protein molecular weight standard (Precision Plus Protein™ Dual Xtra Prestained Protein Standards 2-250 kD, Bio-Rad Laboratories, Inc., Hercules, CA, USA). Separated proteins were fixed in a gel fixing solution containing 50% (*v*/*v*) ethanol and 10% (*v*/*v*) acetic acid then washed with a solution consisting of 50% (*v*/*v*) methanol and 10% (*v*/*v*) acetic acid. Following washing, gel staining was performed using Coomassie blue R-250 solution at room temperature for 3 h with gentle agitation. (Bio-Rad Laboratories, Inc., Hertfordshire, UK), then the gel was destained with washing solution until the background color was removed.

### 2.7. Fractionation Using Ultrafiltration

Green crab hydrolysates were fractionated using ultrafiltration based on molecular weight. A volume of 15 mL of solubilized sample (50 mg/mL) was added into a 30 kD molecular weight cut-off (MWCO) filter device (Amicon^®^ ultra-15 centrifugal filters, EMD Millipore, Burlington, MA. USA) for the first fraction, then centrifuged at 3234× *g* until the volume of retentate reached 250 µL. After collecting the retentate, the <30 kD filtrate was transferred to a 10 kD MWCO filter device for the second fraction. After centrifugation at 3234× *g*, 250 µL of retentate was collected. The <10 kD filtrate was added to the 3 kD MWCO filter device for the third fractionation and was centrifuged at 3234× *g* until the retentate volume reached 250 µL. Both the retentate and the <3 kD fraction were collected and all the hydrolysate fractions were stored at −80 °C until used for further assays.

### 2.8. Rat Intestine α-Glucosidase Inhibition Assay

Rat intestine α-glucosidase inhibition assay was conducted according to the protocol in the Worthington Enzyme Manual with modifications [32] and Kwon et al. [33]. Crude enzyme was extracted from rat intestine acetone powder. For the extraction, 0.3 g of rat intestinal acetone powder was added to 12 mL of 0.1 M sodium phosphate buffer (pH 6.9 with 0.9% NaCl), then sonicated 12 times in 30 s pulses. After centrifugation at 10,000× *g* for 30 min at 4 °C, the supernatant was used as the enzyme solution. A volume of 50 µL of solubilized sample or acarbose (positive control) and 100 µL of enzyme solution were added in a 96 well plate then incubated at 37 °C for 10 min. Then, 50 µL of 5 mM p-NPG solution in 0.1 M phosphate buffer (pH 6.9 with 0.9% NaCl) was added and the mixture was incubated at 37 °C for 30 min. The absorbance was measured at 405 nm by a microplate reader (Ex 808, Biotek, Winooski, VT, USA) and compared with a control containing 50 µL of 0.1 M sodium phosphate buffer in place of the sample. The α-glucosidase inhibitory activity was calculated as follows:

(4) $$\%\;\mathrm{inhibition}=\frac{\left(\Delta A_{405}^{\mathrm{Control}}-\Delta A_{405}^{\mathrm{sample}}\right)}{\Delta A_{405}^{\mathrm{Control}}}\times 100$$

### 2.9. Porcine Pancreatic α-Amylase Inhibition Assay

The α-amylase inhibitory activity was evaluated following a modified version of the assay described by the Worthington Enzyme Manual [34] and Kwon et al. [26]. Porcine pancreatic α-amylase solution (100 unit/mL) was prepared in 0.02 M sodium phosphate buffer (pH 6.9 with 6 mM NaCl). A volume of 100 µL of solubilized sample or acarbose (positive control) and 100 µL of enzyme solution was incubated at 25 °C for 10 min, then 100 µL of 1% starch solution in 0.02 M sodium phosphate buffer (pH 6.9 with 6 mM NaCl) was added. After incubation at 25 °C for 10 min, 200 µL of dinitrosalicylic acid color reagent was added, and, subsequently, the mixture was boiled in a water bath for 5 min. After cooling, 50 µL of the mixture was diluted with 200 µL of deionized water and the absorbance was measured at 540 nm by a microplate reader (Ex 808, Biotek, Winooski, VT, USA). The measured absorbance was compared with a control containing 100 µL of 0.02 M sodium phosphate buffer in place of the sample and the α-amylase inhibitory activity was calculated as follows:

(5) $$\%\;\mathrm{inhibition}=\frac{\left(\Delta A_{540}^{\mathrm{Control}}-\Delta A_{540}^{\mathrm{sample}}\right)}{\Delta A_{540}^{\mathrm{Control}}}\times 100$$

### 2.10. DPP-IV Inhibition Assay

The DPP-IV inhibitory activity was determined using a method from Li-Chan et al. [35]. DPP-IV enzyme was purchased from EMD Millipore (Burlington, MA, USA) and 0.01 unit/mL of enzyme solution was prepared in 100 mM Tris-HCl buffer (pH 8.0). A total of 25 µL of sample and sitagliptin (positive control) and 25 µL of 1.59 mM Gly-Pro-p-nitroanilide solution in 100 mM Tris-HCl buffer (pH 8.0) was preincubated at 37 °C for 10 min, then 50 µL of enzyme solution was added. After incubation at 37 °C for 60 min, 100 µL of 1 M sodium acetate solution was added to stop the reaction. The absorbance was determined at 405 nm by a microplate reader (Ex 808, Biotek, Winooski, VT, USA). The absorbance was compared with a control of 25 µL of 100 mM Tris-HCl buffer (pH 8.0) instead of the sample solution. The DPP-IV inhibitory activity was expressed as percentage inhibition and was calculated as follows:

(6) $$\%\;\mathrm{inhibition}=\frac{\left(\Delta A_{405}^{\mathrm{Control}}-\Delta A_{405}^{\mathrm{sample}}\right)}{\Delta A_{405}^{\mathrm{Control}}}\times 100$$

### 2.11. GLUTag Cell Culture

Murine GLUTag cells that are widely used for the stimulation of GLP-1 secretion were used in this study. GLUTag cells were gifted from Dr. D.J. Drucker (University of Toronto, Toronto, Canada) and cultured in Dulbecco’s Modified Eagle Medium (DMEM, Invitrogen, Carlsbad, CA, USA) containing 5.5 mM glucose (pH 7.4) supplemented with 10% (*v*/*v*) fetal bovine serum, 100 U/mL penicillin-streptomycin, and 2 mM L-glutamine in a 75 cm^2^ culture flask at 5% CO_2_ and 37 °C and GLUTag cells at 80% confluency were regularly trypsinized for subculture.

### 2.12. Cell Viability Assay

Viability of the GLUTag cells was evaluated using the MTT (3-(4,5-dimethylthiazol-2-yl)-2,5-diphenyl tetrazolium bromide) assay. The surface of 96-well culture plates was coated with poly-D-lysine solution. A density of 2 × 10^4^ cells was seeded in each well and incubated at 37 °C, 5% CO_2_, and 80% humidity for 48 h. A volume of 20 µL sample solution (final concentration: 0.2–10 mg/mL) was added to the cells and incubated for 24 h. Then, the mixture was replaced with 100 µL of 0.5 mg/mL MTT reagent and incubated at 37 °C and 5% CO_2_ for 4 h. The MTT reagent was removed, then the precipitate was solubilized in 100 µL of DMSO. The absorbance was measured at 540 nm by a microplate reader (Ex 808, Biotek, Winooski, VT, USA) and compared with a control containing 20 µL of ultrapure water in place of the sample solution. The cell viability was calculated as follows:

(7) $$\%\;\mathrm{cell}\;\mathrm{viability}=\frac{\left(\Delta A_{540}^{\mathrm{Sample}}-\Delta A_{540}^{\mathrm{blank}}\right)}{\left(\Delta A_{540}^{\mathrm{Control}}-\Delta A_{540}^{\mathrm{blank}}\right)}\times 100$$

### 2.13. GLP-1 Secretion Assay

GLP-1 secretory activity was evaluated by an assay modified from Ojo et al. [36]. GLUTag cells were seeded at a density of 2 × 10^5^ in 24-well culture plates coated with poly-D-lysine and grown for 48 h in a 37 °C and 5% CO_2_ incubator until the confluency reached 80%. The cells were washed with phosphate-buffered saline (PBS), then 1 mL of Kreb Ringer Buffer (KRB) containing 115 mM NaCl, 4.7 mM KCl, 1.28 mM CaCl_2_, 1.2 mM MgSO_4_, 1.2 mM KH_2_PO_4_, 25 mM HEPES, and 1M NaHCO_3_ supplemented with 1% bovine serum albumin (BSA, fatty-acid-free) and 1 mM glucose (pH 7.4) was added. After pre-incubation at 37 °C and 5% CO_2_ for 45 min, the KRB was replaced with 1 mL of appropriate KRB test solutions containing sample or 10 mM L-glutamine (positive control). All the KRB test solutions were prepared with supplementation of 1% BSA and 2 mM glucose (pH 7.4). The culture plates were incubated at 37 °C and 5% CO_2_ for 2 h, then the mixture was collected. After centrifugation at 4 °C and 800× *g* for 5 min, the supernatant was collected and stored at −80 °C until further evaluation of GLP-1 concentration. Total GLP-1 concentration was determined based on the manufacturer’s instructions in a commercial ELISA kit (GLP-1 Total ELISA, Millipore, Burlington, MA, USA). The GLP-1 secretory activity was expressed as a percentage (%) of the negative control (KRB buffer).

### 2.14. Statistical Analysis

The enzymatic hydrolysis process using each of the four commercial proteases was replicated three times and all of the assays were conducted in triplicate on each sample replicate. Statistical differences among the means of each treatment were evaluated using one-way analysis of variance (ANOVA) followed by Tukey’s HSD post hoc test and paired *t*-test with a significance value of *p* < 0.05 (SPSS ver. 23, IBM Corp., Armonk, NY, USA). Correlations (*p* < 0.05) between the degree of hydrolysis (DH) and each biofunctional activity were analyzed through the Pearson coefficient (SPSS ver. 23, IBM Corp., Armonk, NY, USA).

## 3. Results

### 3.1. Degree of Hydrolysis

**Figure 2:**
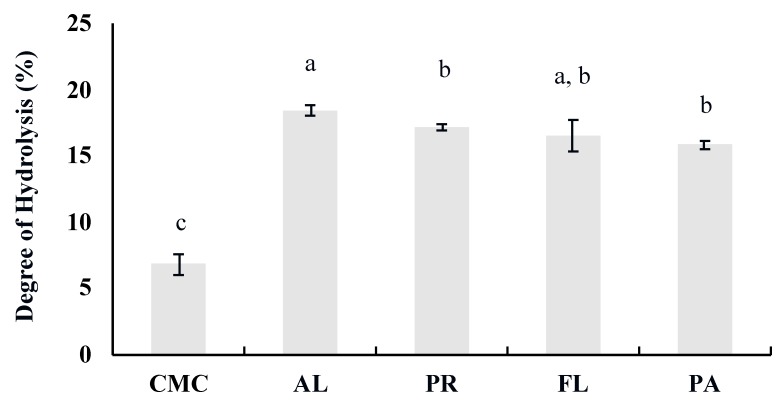
Degree of hydrolysis of green crab mince by enzyme type. CMC: Crab Mince Control, AL: Alcalase, PR: Protamex, FL: Flavourzyme, and PA: Papain. Each bar indicates the mean and standard deviation. Different letters on the bars indicate a significant difference (*p* < 0.05) among the treatments (*n* = 3).

The degree of hydrolysis (DH) was determined by the OPA method with serine as a standard. DH was significantly increased after enzymatic hydrolysis for 60 min. DH of green crab hydrolysates ranged from 15.8% to 18.4%, while DH of the CMC (crab mince control) was 6.8%. Among the treatments, AL showed the highest DH (18.4% ± 0.4%) followed by the PR (17.1% ± 0.2%), FL (16.5% ± 1.2%), and PA (15.8% ± 0.3%) treatments, respectively (Figure 2).

### 3.2. Molecular Weight Distribution Using SDS-PAGE

**Figure 3:**
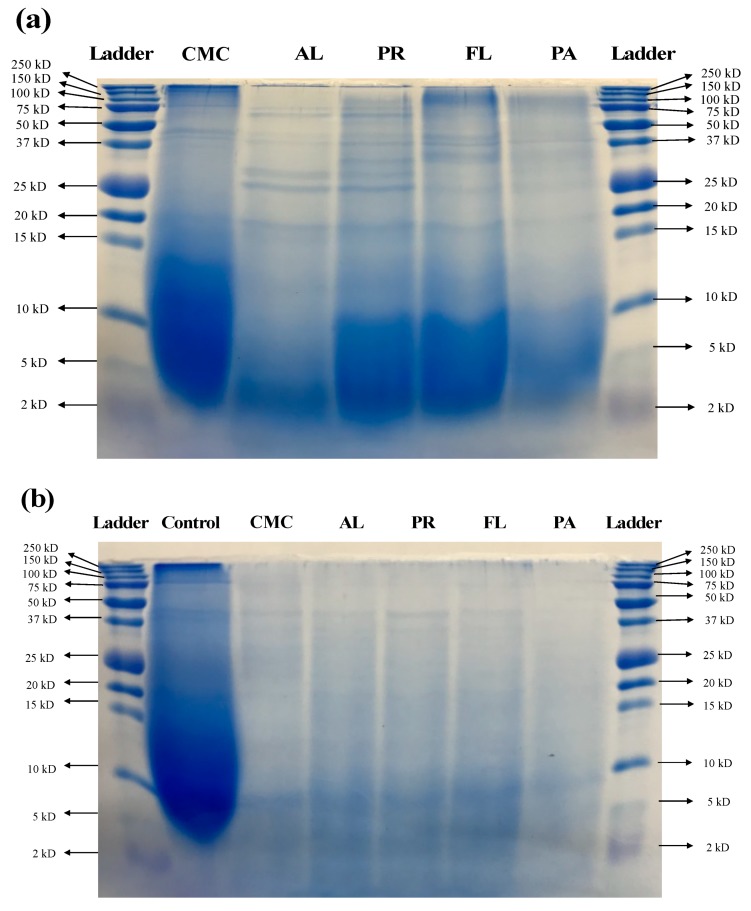
Molecular weight distribution (SDS-PAGE) of CMC and hydrolysates before and after simulated digestion. (**a**) Before simulated digestion. Lane 1 and 7: molecular weight ladder. Lanes 2–6: CMC, AL-derived hydrolysate, PR-derived hydrolysate, FL-derived hydrolysate, and PA-derived hydrolysate, respectively. (**b**) After simulated digestion. Lanes 1 and 8: molecular weight ladder. Lanes 2–7: simulated digestion process control, CMC, AL-derived hydrolysate, PR-derived hydrolysate, FL-derived hydrolysate, and PA-derived hydrolysate, respectively. CMC: Crab Mince Control, AL: Alcalase, PR: Protamex, FL: Flavourzyme, and PA: Papain.

The size distribution of proteins and peptides in the CMC and hydrolysates was determined using SDS-PAGE (Figure 3a). Significant hydrolysis was observed in all of the treatments compared to the CMC. Heavy chains in the 150–250 kD range were reduced by all the enzyme treatments. There were thick bands between 100 kD and 75 kD in the hydrolysates derived by FL and PA, and all of the treatments produced various bands between 20 kD and 37 kD. Similar band patterns were observed in the AL and PR treatments while FL and PA treatments resulted in similar molecular weight profiles. AL-derived hydrolysate showed the lowest intensity of bands between 2 and 10 kD compared to the CMC and other hydrolysates. During simulated digestion, the CMC was hydrolyzed by digestive enzymes as compared to the process control that was applied to the simulated digestion with no digestive enzymes (Figure 3b). The simulated digestion process further hydrolyzed all the hydrolysates obtained from commercial enzyme treatments. The intensity of bands above 15 kD decreased significantly as compared to before the simulated digestion, and the intensity of observed bands at <10 kD also decreased after the simulated digestion.

### 3.3. α-Glucosidase and Porcine α-Amylase Inhibitory Activities

**Figure 4:**
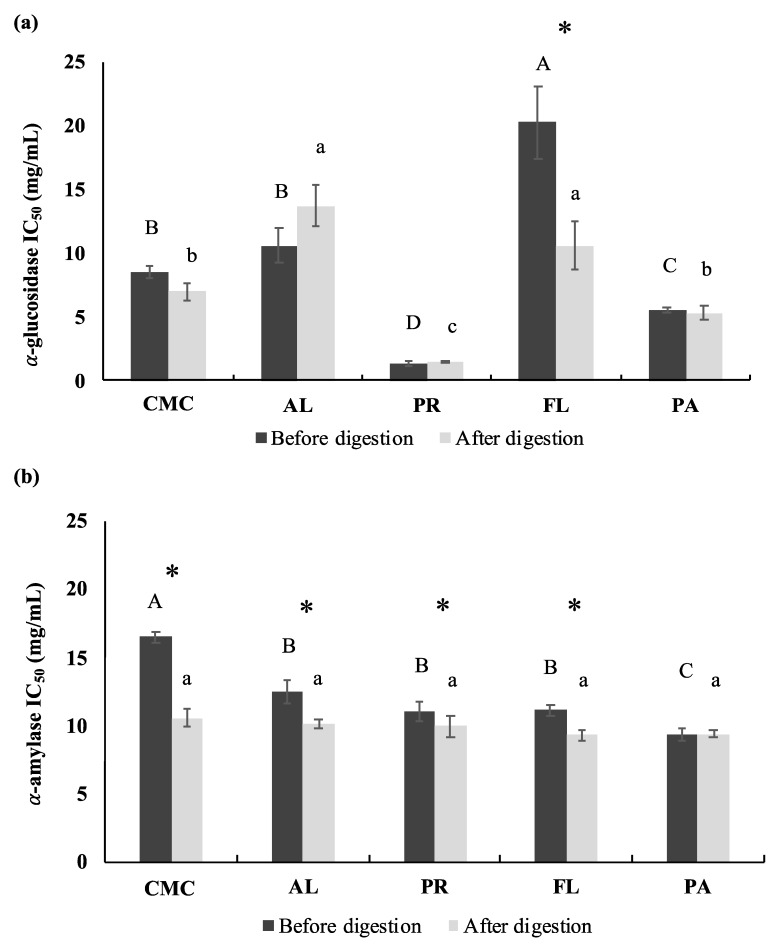
Rat intestinal α-glucosidase and porcine α-amylase inhibitory activities of CMC and hydrolysates before and after simulated digestion. (**a**) Rat intestinal α-glucosidase inhibitory activity. (**b**) Porcine α-amylase inhibitory activity. The IC_50_ values were represented by final assay concentration (protein and peptide basis). CMC: Crab Mince Control, AL: Alcalase, PR: Protamex, FL: Flavourzyme, and PA: Papain. Each bar indicates the mean and standard deviation (*n* = 3 treatment replicates). The letters on the bars indicate a significant difference (*p* < 0.05) among the treatments. An asterisk (*) represents significant difference after simulated digestion (*p* < 0.05) by paired *t*-test.

The inhibitory activity of rat intestinal α-glucosidase and porcine pancreatic α-glucosidase was assessed to evaluate the potential anti-hyperglycemic effect of the CMC and crab hydrolysates. IC_50_ values of both α-glucosidase and α-amylase were determined to evaluate the efficacy of the inhibition. Acarbose was used as a positive control. Acarbose had IC_50_ values of 0.027 mg/mL and 0.84 mg/mL for α-glucosidase and α-glucosidase inhibitory activity, respectively. The α-glucosidase inhibitory activity of the CMC was significantly improved by the PR (IC_50_ 1.38 ± 0.19 mg/mL) and PA (IC_50_ 5.56 ± 0.19 mg/mL) treatments while the FL (IC_50_ 20.24 ± 0.19 mg/mL) treatment showed a decrease in α-glucosidase inhibitory activity compared to the CMC (IC_50_ 8.54 ± 0.50 mg/mL) (Figure 4a). Simulated digestion did not significantly affect the α-glucosidase inhibitory activity of hydrolysates obtained from the PR (IC_50_ 1.49 ± 0.08 mg/mL) and PA (IC_50_ 5.31 ± 0.51 mg/mL) treatments. However, the inhibitory activity of the FL-derived hydrolysate was significantly improved after the simulated digestion (IC_50_ 10.61 ± 1.90 mg/mL). The green crab hydrolysates obtained from PR showed the highest α-glucosidase inhibitory activity before and after simulated digestion and the activity was approximately 4–14 times higher compared to other enzyme treatments and CMC based on IC_50_ values.

The porcine pancreatic α-amylase inhibitory activity of the CMC was statistically increased by enzymatic hydrolysis using commercial enzymes. Among the treatments, PA (IC_50_ 9.35 ± 0.42 mg/mL) exhibited the highest α-amylase inhibitory activity followed by PR (IC_50_ 11.02 ± 0.69 mg/mL), FL (IC_50_ 11.12 ± 0.37 mg/mL), AL (IC_50_ 12.53 ± 0.85 mg/mL), and the CMC (IC_50_ 16.49 ± 0.41 mg/mL), respectively (Figure 4b). Digestive enzymes including pepsin and pancreatin significantly improved the α-amylase inhibitory activity of CMC (IC_50_ 10.55 ± 0.68 mg/mL) and its hydrolysates treated with AL (IC_50_ 10.11 ± 0.34 mg/mL), PR (IC_50_ 9.93 ± 0.82 mg/mL), and FL (IC_50_ 9.31 ± 0.37 mg/mL), however, the inhibitory activity of PA-derived hydrolysate was not affected by simulated digestion. After simulated digestion, the α-amylase inhibitory activity among the treatments was not significantly different.

### 3.4. DPP-IV Inhibitory Activity

**Figure 5:**
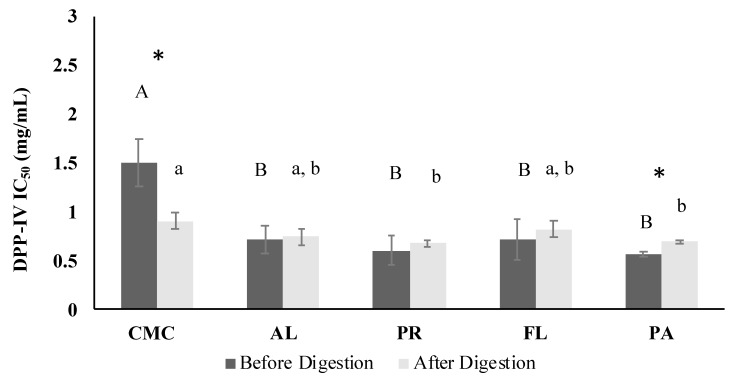
Dipeptidyl peptidase-IV (DPP-IV) IC_50_ values of CMC and hydrolysates. The IC_50_ values were represented by the final assay concentration (protein and peptide basis). CMC: Crab Mince Control, AL: Alcalase, PR: Protamex, FL: Flavourzyme, and PA: Papain. Each bar indicates the mean and standard deviation (*n* = 3 treatment replicates). The letters on the bars indicate a significant difference (*p* < 0.05) among the treatments. An asterisk (*) represents a significant difference after simulated digestion (*p* < 0.05) by paired *t*-test.

Overall, DPP-IV inhibition of CMC (IC50 1.50 ± 0.25 mg/mL) was significantly improved by the enzymatic hydrolysis, and the DPP-IV inhibitory activity IC50 values of hydrolysates ranged from 0.56 mg/mL to 0.72 mg/mL (Figure 5). Sitagliptin was used as a positive control, and had an IC_50_ value of 43.7 ng/mL. The simulated gastrointestinal digestion remarkably enhanced the DPP-IV inhibitory activity of CMC. However, the PR and Papain-treated samples showed significantly higher DPP-IV inhibitory activity than CMC after the simulated digestion.

### 3.5. GLP-1 Secretory Activity

**Figure 6:**
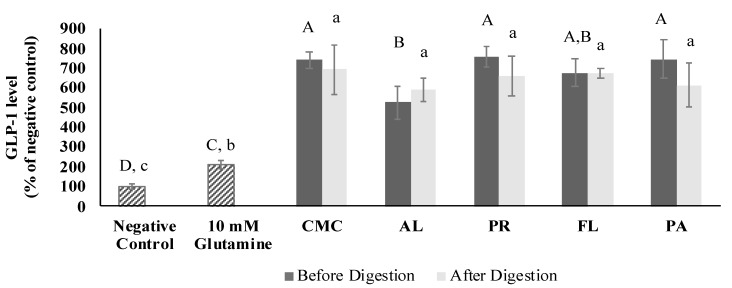
Glucagon-like 1 (GLP-1) level of CMC and hydrolysates before and after simulated digestion determined using GLUTag cells. The GLP-1 level was measured using 0.50 mg/mL of samples (final assay concentration, protein, and peptide basis). CMC: Crab Mince Control, AL: Alcalase, PR: Protamex, FL: Flavourzyme, and PA: Papain. Each bar indicates the mean and standard deviation (*n* = 3 treatment replicates). The letters on the bars indicate a significant difference (*p* < 0.05) among the treatments.

The GLP-1 secretory activity was measured at a sample concentration of 0.5 mg/mL. The cytotoxicity of the CMC and hydrolysates was determined using the MTT assay before evaluating their GLP-1 secretory activity. As a result, there was no observed cytotoxicity on GLUTag cells at 0.125, 0.25, and 0.5 mg/mL (Figure S1). The GLP-1 levels induced by the CMC and crab hydrolysate treatments were evaluated using the GLUTag cell model. Next, the results were compared with glutamine as a positive control (Figure 6). The GLP-1 secretion induced by the CMC and hydrolysates was significantly higher than that by the positive control by 2.5–3.5 times. Among the samples, AL-derived hydrolysate showed the lowest GLP-1 secretory activity, and the activity of all the samples was not significantly affected by simulated gastrointestinal digestion.

### 3.6. α-Glucosidase Inhibitory Activity of Fractions

**Figure 7:**
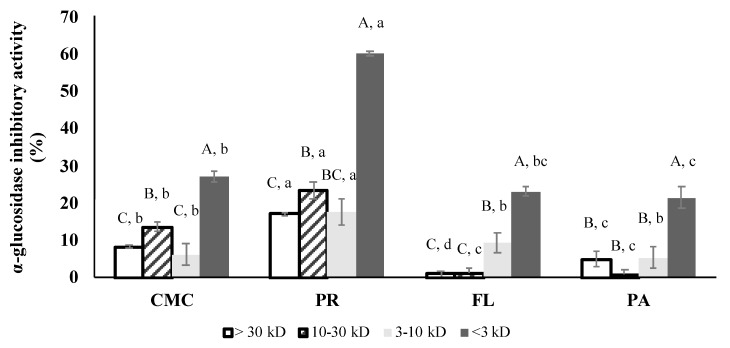
Rat intestinal α-glucosidase inhibitory activities of CMC and hydrolysates after fractionation (final assay concentration: 3.2 mg/mL protein and peptide basis). CMC: Crab Mince Control, AL: Alcalase, PR: Protamex, FL: Flavourzyme, and PA: Papain. Each bar indicates the mean and standard deviation (*n* = 3 treatment replicates). Uppercase letters on the bars indicate a significant difference (*p* < 0.05) within each treatment. Lowercase letters on the bars indicate a significant difference (*p* < 0.05) among the treatments.

Based on the positive results of their α-glucosidase and GLP-1 secretory activities, PR, FL, and PA were selected for subsequent fractionation. Figure 7 represents the α-glucosidase inhibitory activity of the fractions determined at 3.2 mg/mL. PR-treated fractions exhibited the highest α-glucosidase inhibitory activity among the treatments. Within the fractions, the highest α-glucosidase inhibitory activity was shown in the <3 kD fractions of all of the treatments. The <3 kD fraction of the PR-derived hydrolysate inhibited the α-glucosidase activity by 60% while the α-glucosidase inhibitory activity of other fractions was less than 50%. IC_50_ values could only be calculated for the <3 kD fraction since the highest inhibitory activity of the >3 kD fractions was less than 50%. The PR treatment (IC_50_ 1.75 ± 0.24 mg/mL) resulted in the highest α-glucosidase inhibitory effect followed by PA, CMC, and FL.

### 3.7. α-Amylase Inhibitory Activity of Fractions

**Figure 8:**
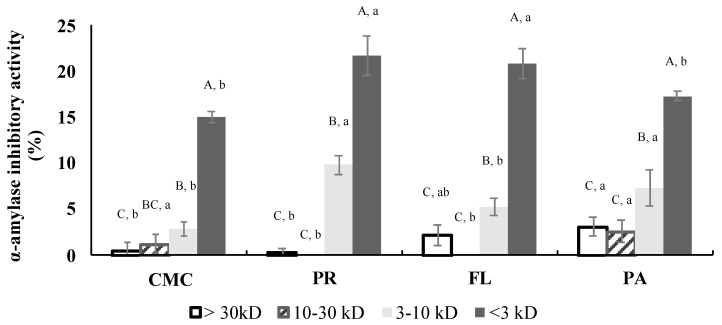
Porcine pancreatic α-amylase inhibitory activities of CMC and hydrolysates after fractionation (final assay concentration: 4.2 mg/mL protein and peptide basis). CMC: Crab Mince Control, AL: Alcalase, PR: Protamex, FL: Flavourzyme, and PA: Papain. Each bar indicates the mean and standard deviation (*n* = 3 treatment replicates). Uppercase letters on the bars indicate a significant difference (*p* < 0.05) within each treatment. Lowercase letters on the bars indicate a significant difference (*p* < 0.05) among the treatments.

The α-amylase inhibition of all the fractions was evaluated at 4.2 mg/mL (Figure 8). The <3 kD fractions had the highest inhibitory activity among the fractions. The inhibitory activities of the <3 kD fractions were not significantly different between PR and FL treatments, which were significantly higher than the α-amylase inhibitory activities of the <3 kD fractions of CMC and PA-derived hydrolysates. IC_50_ values of all the <3 kD fractions ranged from 8.36 to 10.88 mg/mL and were not significantly different among the treatments.

### 3.8. DPP-IV Inhibitory Activity of Fractions

**Figure 9:**
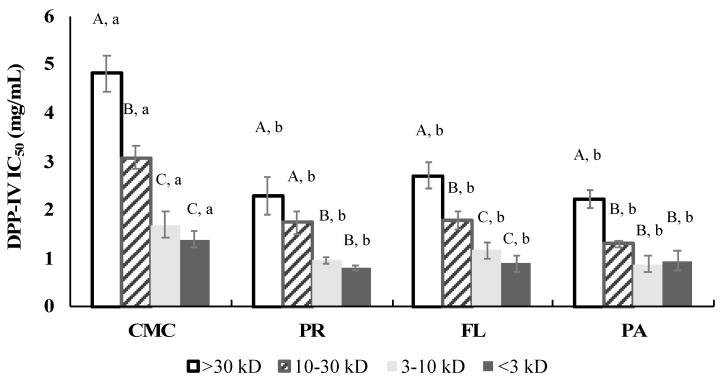
DPP-IV IC_50_ values of CMC and hydrolysates after fractionation. The IC_50_ values were represented by final assay concentration (protein and peptide basis). CMC: Crab Mince Control, AL: Alcalase, PR: Protamex, FL: Flavourzyme, and PA: Papain. Each bar indicates the mean and standard deviation (*n* = 3 treatment replicates). Uppercase letters on the bars indicate a significant difference (*p* < 0.05) within each treatment. Lowercase letters on the bars indicate a significant difference (*p* < 0.05) among the treatments.

The DPP-IV inhibitory ability of the fractions obtained from the fractionation of the ±CMC and hydrolysates is shown in Figure 9. According to IC_50_ values, the <3 kD fraction (CMC: 1.39 ± 0.16 mg/mL, PR: 0.79 ± 0.05 mg/mL, FL: 0.88 ± 0.17 mg/mL, and PA: 0.93 ± 0.20 mg/mL) and 3–10 kD fraction (CMC: 1.68 ± 0.28 mg/mL, PR: 0.95 ± 0.06 mg/mL, FL: 1.15 ± 0.16 mg/mL, and PA:0.88 ± 0.17 mg/mL) within each sample showed the highest DPP-IV inhibitory activity while the > 30 kD fraction (CMC: 4.80 ± 0.37 mg/mL, PR: 2.29 ± 0.40 mg/mL, FL: 2.70 ± 0.26 mg/mL, and PA: 2.21 ± 0.20 mg/mL) exhibited the lowest DPP-IV inhibitory activity within enzyme treatments. The DPP-IV inhibitory activity of all the fractions was improved after the commercial enzyme treatments; however, there was no significant difference among the treatments.

### 3.9. GLP-1 Secretory Activity of Fractions

**Figure 10:**
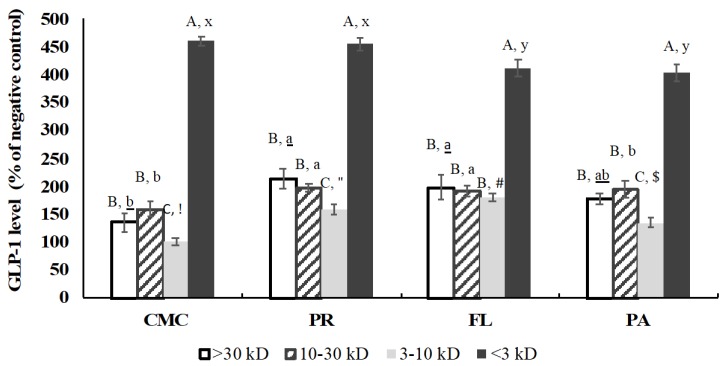
GLP-1 level of CMC and hydrolysates after fractionation (final assay concentration: 0.5 mg/mL protein and peptide basis). CMC: Crab Mince Control, AL: Alcalase, PR: Protamex, FL: Flavourzyme, and PA: Papain. Each bar indicates the mean and standard deviation (*n* = 3 treatment replicates). Lowercase, lowercase letters with underline, and Greek letters on the bars indicate a significant difference (*p* < 0.05) within each treatment. Uppercase letters on the bars indicate a significant difference (*p* < 0.05) among the treatments.

After the fractionation, the total GLP-1 level induced by the CMC and crab hydrolysates (0.5 mg/mL) was evaluated using GLUTag cells. The GLP-1 secretory activity of fractions was improved by the enzymatic hydrolysis except for the >30 kD fraction of PA-derived hydrolysate and <3 kD fractions of FL and PA-derived hydrolysates (Figure 10). Glutamine (10 mM, positive control) increased GLP-1 level by 216% ± 13% compared to a negative control (KRB buffer), and the <3 kD fractions had significantly higher GLP-1 levels than the positive control.

## 4. Discussion

In recent years, as interest in healthy lifestyles has increased, the demand for healthy diets, natural food ingredients, and bioactive compounds has intensified in consumers as well as the food industry. Proteins and peptides are being widely used as nutraceutical food ingredients in food products and dietary supplements to enhance human health and product value. Many bioactive peptides having antioxidant, anti-hypertensive, and anti-hyperglycemic effects have been derived from enzymatic hydrolysis of protein sources including meat, dairy, fish, and their byproducts [15,16,17,18,19,20,37,38,39]. However, there has been limited investigation into bioactive peptides derived from molluscan and crustacean resources.

The bioactive properties of peptides obtained by enzymatic hydrolysis are influenced by (1) the primary amino acid sequence of the protein source, (2) the specificity of the proteases applied, and (3) process parameters such as DH, hydrolysis duration, and enzyme:substrate ratio. As a primary protein source, underutilized green crab was used, which contains ~80% protein in its meat [7]. Manual removal of green crab meat from the carapace is extremely labor-intensive because of the crab’s very small size. Therefore, in this study, a mechanical separator was used to generate green crab meat mince that was subsequently used as the substrate for the enzymatic hydrolysis. To apply proteases with various specificity to the crab homogenate, four commercially available proteases that have two different modes of action were selected. AL, PR, and PA are endopeptidases, while FL has both endo- and exopeptidase characteristics. After hydrolysis, the DH was determined based on the number of cleaved peptide bonds over the total number of peptide bonds. Based on our results, DH significantly increased in response to enzyme treatment (Figure 2). According to Jamdar et al. [40], the bioactivity of protein hydrolysates was modified as DH was changed within the same enzyme treatment. Therefore, the determination of DH is necessary for controlling the enzymatic hydrolysis process to reproducibly obtain bioactive peptides.

In addition to the increase in DH, the molecular weight distribution of the CMC and hydrolysates before the simulated digestion also confirms that the enzymatic process was successful, and that different products were generated based on the commercial protease applied (Figure 2 and Figure 3). Although similar MW patterns were observed in AL and PR-treated samples, their activities were significantly different in terms of α-glucosidase inhibition and GLP-1 secretion (Figure 3 and Figure 5). The weak intensity of AL-derived hydrolysate bands between 2 and 10 kD may be associated with its DH (Figure 3a). In many studies, AL showed an outstanding ability to hydrolyze large proteins to small peptides, and produced smaller peptides more rapidly compared to other commercially available proteases [41,42]. Therefore, the lower intensity of the 2–10 kD bands in the AL treatment was most likely due to a large number of peptides smaller than <2 kD passing through the gel during the SDS-PAGE. After the simulated digestion, the amount of larger size peptides was decreased, and the overall intensity of all the bands became weaker (Figure 3a,b). This demonstrates that proteins and peptides in the CMC and hydrolysates were further hydrolyzed by the digestive enzymes and suggests that the smaller sized peptides may have passed through the gel during electrophoresis.

Pancreatic α-amylase and intestinal α-glucosidase are the two key enzymes involved in starch digestion, resulting in an increase in blood glucose levels. Therefore, the inhibition of both carbohydrases can be an indicator of the potential anti-hyperglycemic effect [43]. According to the DH and SDS-PAGE results, AL hydrolyzed the green crab protein to a greater degree compared to the other enzymes (Figure 2 and Figure 3a). However, results indicate that PR generated the most effective α-glucosidase inhibitive hydrolysates (Figure 4), and that the inhibitory activity was not statistically correlated (*p* > 0.05) with DH. The IC_50_ values of the control and the hydrolysates were strongly correlated with the <3 kD fractions (*r* = 0.987, *p* < 0.05), which indicates that the peptides in the <3 kD fraction are key contributors to the α-glucosidase inhibitory activity. The α-glucosidase inhibitory activity of PR-treated sample (IC_50_ 1.38 ± 0.19 mg/mL) was remarkably higher than not only other treatments and the control but also the hydrolysates of sardine muscle (IC_50_ 48.7 mg/mL), whey protein isolate (IC_50_ 4.5 mg/mL), and edible insects (IC_50_ > 2.0 mg/mL) including mealworm larvae, crickets, and silkworm pupae [38,44,45]. In many studies, AL, FL, and digestive enzymes including pepsin and trypsin are commonly used to obtain bioactive peptides [38,39,45,46]. Interestingly, in the current study, AL and FL treatment did not show improved inhibitory activity compared to the control. The high α-glucosidase inhibitory activity of the PR-treated sample emphasizes the importance of protein source and protease selection in obtaining bioactive peptides. A major concern regarding the use of bioactive peptides for human consumption and industrial application are the changes in biological activity as a result of further hydrolysis during gastrointestinal digestion and processing. However, simulated digestion did not affect the α-glucosidase inhibitory activities of PR and PA-treated samples, suggesting that their inhibitory activity was stable to pepsin and pancreatin action during the gastrointestinal digestion.

The enzymatic hydrolysis by commercial enzymes, and subsequently by digestive enzymes, improved the α-amylase inhibitory activity of the green crab proteins. The inhibitory activity was most likely primarily due to the <3 kD fractions (Figure 4b and Figure 8) since those showed the highest inhibitory activity among the same concentration of fractions. The α-amylase inhibition of the PR-treated sample was weaker than its α-glucosidase inhibition which may contribute to reducing the side-effects that are frequently caused by inhibition of carbohydrate hydrolyzing enzymes. Acarbose, a synthetic pharmaceutical α-glucosidase and α-amylase inhibitor, has been commonly used to inhibit glucose absorption in diabetic patients. However, its strong α-amylase inhibitory activity (IC_50_ <1 mg/mL) leads to the presence of non-digested polysaccharides in the large intestine, which causes side-effects including severe stomach pain, constipation, and diarrhea [47]. Therefore, the use of α-glucosidase and α-amylase inhibitors based on the PR-derived hydrolysates might be a good alternative to delay glucose absorption and help control blood glucose spikes. The inhibitory mechanism of both α-glucosidase and α-amylase by bioactive peptides has not been well characterized. However, recent studies have reported that hydrophobic interactions of non-saccharide compounds that allow them to bind to the carbohydrase active site may contribute to their inhibitory activity [48].

DPP-IV is an enzyme that rapidly metabolizes incretins such as active GLP-1 and gastric inhibitory polypeptide (GIP) hormones. These incretins are important since the hormones help in blood glucose control by insulin secretion [49]. DPP-IV as a postproline hydrolyzing enzyme cleaves dipeptides with X-Pro or X-Ala from the N-terminus of polypeptides [50,51]. In this study, the DPP-IV inhibitory activity of the CMC and hydrolysates was investigated as a potential strategy for T2DM management. Enzyme hydrolysis improved the DPP-IV inhibitory activity regardless of the protease applied, and the activity was not significantly (*p* > 0.05) correlated with DH. Recent studies on DPP-IV inhibitors derived from food sources reported that peptides containing Pro, Ala, and Gly at the P1-position and/or Trp at the N-terminal might have effective DPP-IV inhibitory activity [52]. The type of amino acid residues at the P1, P2, and P1′-positions of peptides may significantly influence their DPP-IV inhibitory activity [53]. The <3 kD and 3–10 kD fractions showed similar IC_50_ values to the hydrolysates before the simulated digestion (Figure 5 and Figure 9). Thus, our study suggests that the fractions <10 kD are likely to play an important role in inhibiting the DPP-IV enzyme. However, overall DPP-IV inhibitory activity of the CMC and hydrolysates (IC_50_ 0.56–1.5 mg/mL) was significantly lower than the medication sitagliptin (IC_50_ 43.7 ng/mL) and other dairy protein and salmon byproduct hydrolysates (IC_50_ < 100 µg/mL) [35,38].

GLP-1 hormone has the ability to reduce blood sugar levels by enhancing the secretion of insulin; however, it has a half-life of only ~1.5 min because of rapid inactivation by DPP-IV [54]. The GLP-1 content secreted from GLUTag cells was not different among the samples except for AL treatment that showed a lower GLP-1 level, and the GLP-1 secretory activity was not correlated with DH. In comparison with the positive control and hydrolysates from blue whiting and salmon skin, green crab hydrolysates released approximately 2.5–3.5 times higher GLP-1 levels. This is considered due to the mixture of certain free amino acids and peptides in the crab hydrolysates. Various amino acids including Gln, Glu, Ala, Ser, Leu, Gly, Asn, and Met have been demonstrated to stimulate GLP-1 secretory activity [39,55,56,57,58]. According to Tolhurst [59], there are two major mechanisms involved in the stimulation of GLP-1 release by Gln on Glutag cells: (1) electrogenic sodium-coupled amino acid uptake resulting in a depolarization of membrane and activation of voltage-gated calcium entry, and (2) elaboration of intracellular cAMP levels. Gameiro et al. [58]. reported that amino acids such as Ala and Gly activate ionotropic glycine receptors, which respond to the various amino acids by generating chloride current. Our previous research showed that the green crab mince contains a high amount of Gln + Glu and Asn + Asp [7], which may have contributed to its higher GLP-1 secretory activity compared to the negative and positive controls. Small peptides including tri- and dipeptides (Leu-Gly-Gly, Gly-Leu, and Gly- Pro) are known to stimulate the secretion of GLP-1 from the in vitro cell model [39,60]. Therefore, the high GLP-1 content induced by the <3 kD fractions within treatments might be due to the concentration of amino acids and small di- or tripeptides after the fractionation.

## 5. Conclusions

Enzymatic hydrolysis was successfully applied to mechanically separated green crab mince to generate a potential anti-hyperglycemic food ingredient. Our results suggest that the anti-hyperglycemic effects of green crab hydrolysates were dependent on the type of protease applied, and not on the degree of hydrolysis. Among the proteases evaluated, Protamex generated products having the highest α-glucosidase inhibitory activity. Furthermore, the fractionation study indicated that the <3 kD peptides primarily contributed to the bioactivities of the hydrolysates. Importantly for their prospective use in novel food products, the bioactivities of the hydrolysates were stable after the simulated gastrointestinal digestion. In conclusion, Protamex was the most effective protease for obtaining anti-hyperglycemic hydrolysates from green crab mince, and the development of bioactive hydrolysates may be a viable route for developing value-added food ingredients from this underutilized marine resource. However, substantial further research including identifying the bioactive compounds in the hydrolysates, evaluating their stability in a food model, and conducting in vivo assessment of their effectiveness is required for their future commercial application in health-promoting foods.

## Supplementary Materials

The following are available online at https://www.mdpi.com/2304-8158/9/3/258/s1, Figure S1: Effect of CMC and hydrolysates on GLUTag cells. Each bar indicates the mean and standard deviation (*n* = 3 treatment replicates). Asterisk (*) represents significant difference between negative control and each sample (*p* < 0.05) by paired *t*-test.
