# Supplementary material for: Anti-Hyperglycemic Effects of Green Crab Hydrolysates Derived by Commercially Available Enzymes
Source: Foods. 2020 Feb 28;9(3):258. doi: 10.3390/foods9030258 (PMC7143835; doi:10.3390/foods9030258)
Supplement: Supplementary file 1 [file foods-09-00258-s001.pdf]

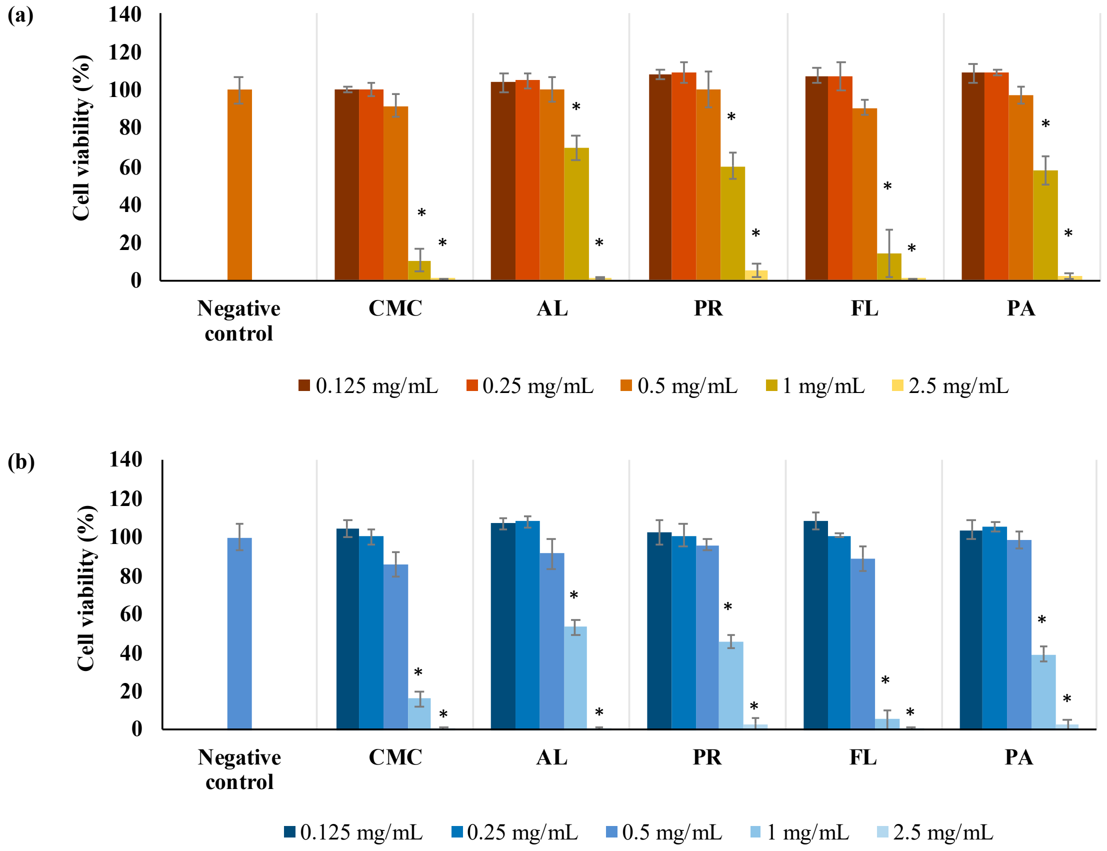

Figure S1. Effect of CMC and hydrolysates on GLUTag cells. Each bar indicates the mean and standard deviation (n = 3 treatment replicates). Asterisk (*) represents significant difference between negative control and each sample ($p < 0.05$) by paired t-test.
